# 16A system of molecular markers to identify alleles of the Rht-B1 and Rht-D1 genes controlling reduced height in bread wheat

**DOI:** 10.18699/VJGB-22-16

**Published:** 2022-03

**Authors:** I.V. Porotnikov, O.P. Mitrofanova, O.Yu. Antonova

**Affiliations:** Federal Research Center the N.I. Vavilov All-Russian Institute of Plant Genetic Resources (VIR), St. Petersburg, Russia; Federal Research Center the N.I. Vavilov All-Russian Institute of Plant Genetic Resources (VIR), St. Petersburg, Russia; Federal Research Center the N.I. Vavilov All-Russian Institute of Plant Genetic Resources (VIR), St. Petersburg, Russia

**Keywords:** Triticum aestivum, alleles of Rht-genes, AS-PCR, CAPS, dCAPS, genotyping, Triticum aestivum, аллели Rht-генов, AS-PCR, dCAPS, CAPS, генотипирование

## Abstract

Mutant alleles of the Rht-B1 and Rht-D1 (Reduced height) genes are widely used in bread wheat breeding for the development of intensive-type cultivars. These genes and their f lanking regions have been sequenced and the point mutations leading to the nonsense codons (Rht-B1b, Rht-B1e, Rht-B1p and Rht-D1b alleles) and various insertions (Rht-B1c, Rht-B1h and Rht-B1i-1) associated with a change in plant height have been described. DNA-markers based on the allele-specif ic PCR have been developed to identify single-nucleotide changes. However, the use of such technique imposes stringent PCR conditions, and the resulting data are not always unambiguous. An alternative can be found in the CAPS technology: it detects differences in sequences by digesting PCR products. In the absence of restrictases capable of digesting DNA at the point mutation site, restriction sites can be introduced into the primer sequence (derived CAPS). The aim of this study was to propose a system of CAPS-, dCAPS- and STS-markers for identifying alleles of the reduced height genes frequently used in breeding programs. Three CAPS have been developed to identify the Rht-B1b, Rht-D1b, Rht-B1p alleles, as well as two dCAPS for Rht-B1b, Rht-B1e. STS-markers for the insertion-containing alleles Rht-B1c, Rht-B1h and Rht-B1i-1 have been selected from publications. The proposed markers were tested during the genotyping of 11 bread wheat accessions from the VIR collection with the abovementioned mutant alleles and the wild-type Rht-B1a and Rht-D1a. The presence of nonsense mutations was also conf irmed by the results of allele-specif ic PCR. This marker system, along with the existing ones, can be used to identify dwarf ing alleles of the Rht-B1 and Rht-D1 genes in bread wheat for genetic screening of accessions from ex situ collections and/or for marker-assisted selection.

## Abstract (ru)

Мутантные аллели генов Rht-B1 и Rht-D1 (Reduced height) широко используют для создания короткостебельных сортов мягкой пшеницы интенсивного типа. Эти гены и фланкирующие их области секвенированы, в последовательностях описаны ассоциированные с изменением высоты растения однонуклеотидные замены, приводящие к образованию нонсенс-кодонов (аллели Rht-B1b, Rht-B1e, Rht-B1p и Rht-D1b), и различные инсерции (аллели Rht-B1c, Rht-B1h и Rht-B1i-1). Для идентификации такого типа однонуклеотидных мутаций разработаны ДНК-маркеры, основанные на принципе аллель-специфичной полимеразной цепной реакции (ПЦР). Однако идентификация аллелей этим методом предъявляет повышенные требования к соблюдению условий реакции, а получаемые результаты не всегда однозначны. Альтернативой может быть CAPS-технология, детектирующая различия в последовательностях путем рестрикции ПЦР-продуктов. В случае отсутствия рестриктаз, способных расщеплять ДНК в месте локализации точковой мутации, рестрикционные сайты могут быть искусственно внесены в последовательность праймера (derived CAPS). Цель настоящей работы – разработать CAPS- и dCAPS-маркеры для выявления замен оснований, подобрать по литературным источникам STS-маркеры для детекции инсерций и тем самым предложить систему молекулярных маркеров для идентификации аллелей генов короткостебельности, часто используемых и перспективных для селекции. Разработано три CAPS-маркера для выявления аллелей Rht-B1b, Rht-D1b, Rht-B1p и два dCAPS-маркера для Rht-B1b и Rht-B1e, предложены программы для их амплификации. По литературным источникам подобраны STS-маркеры аллелей Rht-B1c, Rht-B1h, Rht-B1i-1, содержащих инсерции. Предложенная система маркеров апробирована при генотипировании 11 образцов мягкой пшеницы из коллекции ВИР, несущих вышеуказанные мутантные аллели генов короткостебельности и аллели дикого типа Rht-B1a и Rht-D1a. Наличие нонсенс-мутаций подтверждено также при помощи аллель-специфичной ПЦР. Эта система маркеров наряду с уже существующими может быть использована для идентификации аллелей генов короткостебельности Rht-B1 и Rht-D1 у мягкой пшеницы с целью генетического скрининга образцов ex situ коллекций и/или в маркер-ориентированной селекции.

## Introduction

The development of intensive-type short-stemmed wheat cultivars is considered one of the key success factors in bread wheat breeding, primarily in implementing the Green Revolution initiative in the world’s developing countries (Hedden, 2003; Sukhikh et al., 2021). The decrease in plant height not only entailed higher resistance to lodging, with its favorable effect on the efficiency of mechanized harvesting, but also increased the number of grains per ear and their number per 1 m2, which aggregately led to higher yields (Gale et al., 1985; Youssefian et al., 1992; Evans, 1998).

At least 25 genes controlling plant height in bread wheat (Triticum aestivum L.) and related species were described: they are known as Reduced height – Rht1–Rht25. All these genes are in one way or another associated with the growth hormone gibberellin (McIntosh et al., 2013, 2016, 2018). Some of them, the so-called GA-sensitive genes Rht4–Rht9, Rht12–Rht20 and Rht25, are apparently involved in the synthesis or degradation of gibberellic acid (GA). Other genes, GA-insensitive ones, such as Rht-A1, Rht-B1, and Rht-D1, determine the response to this acid. For some genes (Rht22, Rht23 and Rht24), the nature of their response has not yet been clarified (Sukhikh et al., 2021).

The most widespread among GA-sensitive genes is Rht8, transferred in the early 20th century, together with the closely linked photoperiod insensitivity allele Ppd-D1a of the Ppd gene (response to photoperiod ), from the Japanese cultivar Akakomugi first to Italian and later to many East and South European cultivars (Borojevic K., Borojevic Ks., 2005). This gene does not exert any significant reducing effect on the coleoptile length and, as a consequence, makes it possible to sow seeds to a greater depth, which plays a decisive role in maintaining the viability of seedlings under water deficits or high temperatures (Korzun et al., 1998; Ellis et al., 2004; Divashuk et al., 2013; Grover et al., 2018).

GA-insensitive genes were studied in more detail; they are located on the short arms of chromosomes of homeologous group 4 (Gale, Marshall, 1976; Börner et al., 1996). Dominant alleles of these genes (wild-type) encode DELLA proteins, belonging to the family of GRAS proteins (transcription regulators); at their C-terminus, there is a conservative domain that can bind to other transcription factors and thereby block their function. That is why large amounts of DELLA proteins in cells decelerate plant growth. There is a DELLA domain at the variable N-terminus: it is capable of forming the GA–GID1 complex (gibberellin insensitive dwarf 1, GA receptor). This complex undergoes polyubiquitination and degradation induced by proteasomes. Accordingly, a decrease in the amount of DELLA proteins in cells in the presence of GA reduces their negative effect on plant growth (Peng et al., 1999; Bazhenov et al., 2015; Thomas, 2017; Sukhikh et al., 2021).

A fairly large number of recessive and semi-dominant mutant alleles altering the stem length in different ways have been described for the Rht-B1 and Rht-D1 genes. These alleles have been sequenced; the most thoroughly studied sequences are presented by us in Supplementary material 11. The alleles Rht- B1b (=Rht1), Rht-B1e (=Rht11, =Rht Krasnodari 1), Rht-B1p (=Rht17) and Rht-D1b (=Rht2) were shown to be associated with single-nucleotide substitutions that lead to the formation of premature stop codons (Peng et al., 1999; Ellis et al., 2002; Pearce et al., 2011; Divashuk et al., 2012; Li et al., 2012; Bazhenov et al., 2015). The phenotypic effect of such nonsense mutations varies from moderate (a decrease in plant height by 20–24 % in the presence of Rht-B1b and Rht-D1b alleles) to strong (by 33 and 40 % in the presence of Rht-B1p and Rht-B1e, respectively) (Gale et al., 1985; Sukhikh et al., 2021).

Supplementary materials 1 and 2 are available in the online version of the paper: http://vavilov.elpub.ru/jour/manager/files/Suppl_Porotnikov_Engl.pdf


The alleles Rht-B1h and Rht-B1i-1 have large (over 100 bp) insertions in the 5′ flanking region, while Rht-B1c (=Rht3) is characterized by the presence of an insertion in the 5′ untranslated region identical to that in Rht-B1h and, at the same time, the presence of the Veju retrotransposon in the coding region (Wu et al., 2011; Li et al., 2013; Wen et al., 2013; Lou et al., 2016). Such insertions can lead to the formation of nondegradable proteins, so the growth of mutant plants is constitutively repressed, more significantly than in the case of nonsense mutations in the N-terminal coding region (Wu et al., 2011; Wen et al., 2013). For example, the Rht-B1c allele reduces plant height approximately by 60 % (Flintham, Gale, 1983; Sukhikh et al., 2021). However, insertions can not only reduce but also increase the height of plants (by 10–15 %, compared to the wild type) as, for example, in the case of Rht-B1i-1 (Lou et al., 2016). Besides, the alleles of “strong dwarfing”, Rht-D1c (Rht10) and Rht-D1d (Rht Ai-bian 1a), reducing the height by 60–70 %, were identified in the Rht-D1 gene; they turned out to be multiple copies of the mutant allele Rht-D1b (Pearce et al., 2011). There are also other known alleles of the Rht-B1h–o and Rht-D1e–j genes, associated with either nucleotide changes (missense mutations) or indels. They are identified in a large number of Chinese cultivars using the EcoTILLING method; however, their phenotypic effect has not yet been described. Mutant alleles of the Rht-A1 gene were also identified for the first time in Chinese cultivars (Li et al., 2013).

The alleles most frequently used in breeding programs are Rht-B1b and Rht-D1b. Their source was the Japanese cultivar Norin 10. At the end of the 20th century, more than 70 % of the world’s bread wheat cultivars contained these alleles (Gale et al., 1985; Evans, 1998). Later, however, it was shown that their occurrence depended on the region of the world. Rht-B1b was detected in 36.2 % of bread wheat cultivars from China, and Rht-D1b in 53.4 % (Zhang et al., 2006). Meanwhile, the genotyping of 247 cultivars from the United States and Canada helped to identify these alleles in more than 90 % of them (Guedira et al., 2010). Rht-D1b predominates in the genotypes of European cultivars, while its occurrence in the cultivars registered after 1990 is 49 % (Würschum et al., 2017).

Widespread in Russia are cultivars with the Rht-B1e allele, obtained by mutagenesis in cv. Bezostaya 1; the mutant form is Krasnodarsky Karlik 1 (Lukyanenko, Zhogin, 1974; Rabinovich, 1986). At present, semi-dwarf cultivars (Kroshka, Pobeda 50, Fisht, Palpich, Vostorg, Doka, Tanya, Yesaul, Kalym, Pervitsa, and Grom), homozygous for Rht-B1e alleles, are cultivated both in Russia and the ex-USSR countries on an area of more than 4 million hectares (Divashuk et al., 2012, 2013).

The allele Rht-B1p is also promising for breeding: a stop codon emerges in its DELLA domain due to the substitution of cytosine for thymine at position 178 from the start codon. This mutation causes an up to 30 cm decrease in the height of bread wheat plants, especially as far as the lower internode is concerned, but it does not reduce the length of the ear (Bazhenov et al., 2015).

The sequencing of Rht-B1 and Rht-D1 alleles in various bread wheat cultivars have led to the development of molecular markers for their identification. For example, STS markers were obtained to identify the insertion-containing alleles Rht-B1c, Rht-B1h and Rht-B1i-1 (Pearce et al., 2011; Li et al., 2013; Lou et al., 2016). Markers based on allele-specific PCR (AS-PCR), including real-time AS-PCR, are used to identify the alleles Rht-B1b, Rht-B1e, Rht-B1p and Rht-D1b, carrying single-nucleotide substitutions (Ellis et al., 2002; Pearce et al., 2011; Li et al., 2012; Bazhenov et al., 2015, 2019).

The widespread alleles Rht-B1b and Rht-D1b as well as those of the Rht24 gene are identified on the basis of competitive allele-specific PCR (KASP-markers), offering a possibility to evaluate large numbers of bread wheat accessions at low time costs (Rasheed et al., 2016; Würschum et al., 2017). It should also be mentioned that AS-PCR results strongly depend on the reaction conditions, require several replications of the analysis, and call for strict observance of the author’s protocol, which is not always possible. The KASP analysis, in its turn, requires sophisticated equipment and expensive reagents, which are often unaffordable to small practice-oriented laboratories.

The use of CAPS (cleaved amplified polymorphic sequence) markers can be an alternative to AS-PCR: they are based on the presence of a restriction site in the region with a singlenucleotide mutation (the site is absent in the wild type) or, contrariwise, on the disappearance of the site typical of the wild type in the mutant version (Shavrukov, 2015). If restriction sites are absent at the locations of the analyzed mutations, they can be produced purposefully through designing modified primers, i. e., by the derived CAPS method, or dCAPS (Neff et al., 1998, 2002).

Unlike AS-PCR, the CAPS and dCAPS marker techniques are effortlessly reproducible and do not require stringent PCR conditions, while the results of such analysis are easily interpreted in agarose gels. It is possible to generate markers using the basic PCR equipment. Previously, such markers were developed for the Rht24 dwarfing gene (Tian et al., 2017).

The objective of the present study was to develop CAPS and dCAPS markers for the analysis of single-nucleotide changes in Rht-B1 and Rht-D1, test STS markers for identification of insertions in these genes and, as a result, propose a marker system for identifying the alleles most frequently used in bread wheat breeding.

## Materials and methods

Plant material. Eleven bread wheat accessions from the VIR collection with known alleles of the Rht-B1 and Rht- D1 dwarfing genes (Table 1) served as the material for this study. The cultivars Chinese Spring and Hongdongmai with wild-type alleles Rht-B1a and Rht-D1a were used as controls. Each of the studied accessions was represented in the genotyping process by two or three individual plants as well as by bulk DNA sample, which was isolated from a total of 10–20 genotypes (seedlings).

**Table 1.**
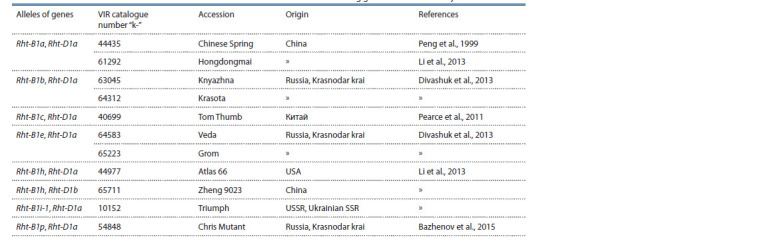
Bread wheat accessions with the known alleles of the Rht-B1 and Rht-D1 dwarf ing genes used in the study

DNA extraction. DNA was extracted from 10-day-old seedlings using a modified CTAB extraction technique (Antonova et al., 2020).

Sequences alignment. The sequences of different alleles of the Rht-B1 and Rht-D1 genes were aligned using MEGA X (https://www.megasoftware.net/), Unipro UGENE (Okonechnikov et al., 2012), and BioEdit Sequence Alignment Editor (Hall, 1999). Restriction sites were searched for using the GenScript Restriction Enzyme Map Analysis Tools (https:// www.genscript.com/tools/restriction-enzyme-map-analysis).

**Fig. 1.**
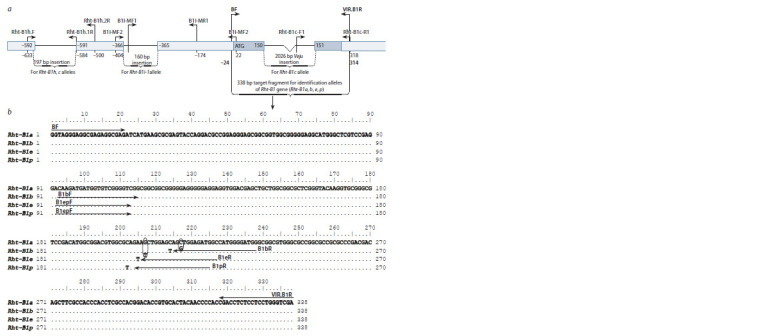
Localization of primers for identifying the main alleles of the Rht-B1 gene. а – scheme of the gene and adjacent regions with the marked primer localizations for the f irst round of nested PCR and for identif ication of insertions in the Rht- B1c, Rht-B1h and Rht-B1i-1 alleles; b – alignment results for the sequences f lanked by the BF/VIR.B1R primers (f irst round of nested PCR), and primers for identif ication of point mutations in the Rht-B1b, Rht-B1e and Rht-B1p alleles using CAPS/dCAPS analysis. Borders of the 197 bp insertion and Veju retrotransposon are taken from the publication by W. Wen et al. (2013); borders of the 160 bp insertion are taken from the publication by X. Lou et al. (2016). The gene’s coding region is f illed with dark color, and the Veju retrotransposon and insertions in the 5’ f lanking region are marked with thin lines. Ovals in Fig. 1, b indicate nucleotide changes in dCAPS primers.

Primers development. Primers for the nested PCR and CAPS analysis were developed with the Primer3Plus software (Untergasser et al., 2007). Primer quality (number of hairpins, homo- and heterodimers) was monitored using OligoAnalyzer Tool, a web resource from Integrated DNA Technologies, Inc. (https://eu.idtdna.com/calc/analyzer). Primers for dCAPS markers were generated using the dCAPS Finder 2.0 software (Neff et al., 2002). The primers developed in the course of this study and those supplied from published sources are presented in Tables 2 and 3, and their locations are shown in Fig. 1, a, b.

**Table 2.**
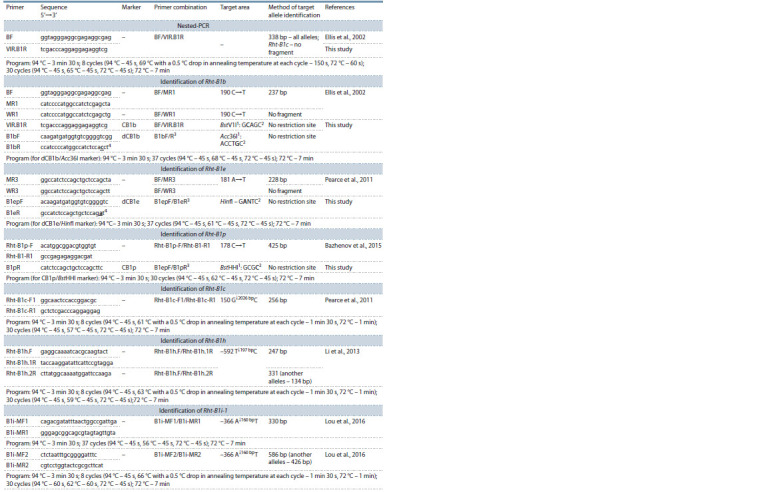
Primers used in this study to identify alleles of the Rht-B1 gene 1 The listed restriction enzymes may be replaced with their isoschizomers: BstV1I (BseXI, BbvI), Acc36I (BveI, BspMI), BstHHI (AspLEI, CfoI, Hin6I, HinP1I, HspAI). 2 Boldfaced in the selective area are the nucleotides at restriction sites, variation of which enables the researcher to identify the required alleles. 3 The nested PCR products of the first round were used as a template for these combinations of primes (50 times dilution). 4 Boldfaced and underlined are the modified nucleotides in primers for dCAPS markers.

**Table 3.**
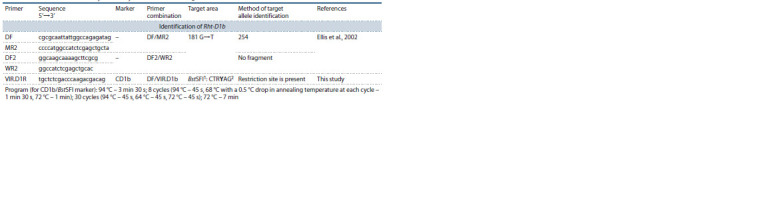
Primers used in this study to identify alleles of the Rht-D1 genes 1 Isoschizomers for the BstSFI restriction enzyme: BfmI, and SfcI. 2 Boldfaced in the selective area are the nucleotides at restriction sites, variation of which enables the researcher to identify the required alleles.

PCR procedure: a) nested PCR. The nested PCR method was applied to enhance the specificity of the dCAPS analysis: the first PCR was performed with primers BF/VIR.B1R flanking the region of point mutations in the Rht-B1 gene; after that, the resulting PCR product was used as a template for the second PCR with dCAPS (B1bF/R, B1epF/B1eR) and CAPS (B1epF/B1pR) primers. The first round of nested PCR was carried out in 25 μl of the reaction mixture containing 40 ng of total wheat DNA; 1× reaction buffer; 1.5 mM of MgCl2; 0.6 mM of each dNTP; 0.25 μM of both forward and reverse primer, and 1 unit of Taq DNA polymerase (Dialat, Russia, http://dialat.ru/). For higher specificity, the PCR program contained the Touchdown function: the initial annealing temperature was 4 degrees higher than the design temperature and decreased by 0.5 degrees per cycle for 8 cycles (see Table 3).

**Fig. 2.**
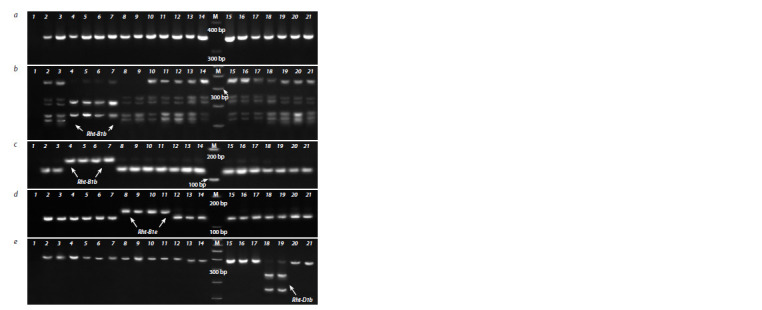
Identification of nonsense mutations in the Rht-B1 and Rht-D1 genes using the developed CAPS and dCAPS markers. a – amplif ication products obtained with the BF/VIR.B1R primers, serving as a template for the second round of nested PCR; b – identif ication of the Rht-B1b allele: CAPS marker CB1b/BstV1I; c – identif ication of the Rht-B1b allele: dCAPS marker dCB1b/ Acc36I; d – identif ication of the Rht-B1e allele: dCAPS marker dCB1e/HinfI; e – identif ication of the Rht-D1b allele: CAPS marker CD1b/BstSFI. Numbers designate accessions with different alleles of the Rht-B1 and Rht-D1 genes: 1 – negative control (H2O); 2, 3 – Hongdongmai (wild-type); 4, 5 – Krasota (Rht-B1b, Rht-D1a); 6, 7 – Knyazhna (Rht-B1b, Rht-D1a); 8, 9 – Grom (Rht-B1e, Rht-D1a); 10, 11 – Veda (Rht- B1e, Rht-D1a); 12, 13 – Chris Mutant (Rht-B1p, Rht-D1a); 14, 15 – Triumph (Rht-B1i-1, Rht-D1a); 16, 17 – Chinese Spring (wildtype); 18, 19 – Zheng 9023 (Rht-B1h, Rht-D1b); 20, 21 – Atlas 66 (Rht-B1h, Rht-D1a). М – molecular marker 100 bp DNA Ladder (SibEnzyme).

Samples of the resulting amplification products (2 μl of each) were transferred into clean tubes, diluted 50 times with water, and used as a template in the second stage of PCR. Another 10 μL of each PCR product was taken to control the success of PCR by agarose gel electrophoresis (Fig. 2, a). The remainder (approximately 12 μl) was treated with the restriction enzyme BstV1I (SibEnzyme, Russia, http://russia. sibenzyme.com/) to generate the CAPS marker for the Rht- B1b allele.

The second round of nested PCR was performed in 20 μl of the reaction mixture containing 4 μl of the template; 1× reaction buffer; 2.5 mM of MgCl2; 0.3 mM of each dNTP; 0.25 μM of both forward and reverse primer, and 1 unit of Taq DNA polymerase (Dialat). The programs for each pair of primers are also presented in Table 3. Approximately 12 μl of the amplification mixture were taken for restriction analysis, and the remainder was used for PCR control by electrophoresis;

b) standard PCR. In the cases of CAPS markers for the Rht-D1b allele and the markers detecting retrotransposon in the gene’s coding region and insertions in the 5′ flanking region, PCR was performed under standard conditions. The reaction mixture (20 μl) contained 40 ng of DNA; 1× reaction buffer; 2.5 mM of MgCl2; 0.3 mM of each dNTP; 0.25 μM of each primer, and 1 unit of Taq DNA polymerase (Dialat); the programs are presented in Tables 2 and 3;

с) allele-specific PCR. The conditions and the programs for AS-PCR corresponded to those recommended by the authors of the primers (Ellis et al., 2002; Bazhenov et al., 2015).

Restriction analysis. PCR products were treated with restriction enzymes produced by SibEnzyme, using the manufacturer’s protocol (http://russia.sibenzyme.com).

Fragment separation was done in horizontal agarose gels in the 1× TBE buffer under the voltage of 5 V/cm. The gels were stained with ethidium bromide and visualized in UV light.

## Results and discussion

For the development of CAPS and dCAPS markers, the sequences from the NSBI databases were analyzed (https:// www.ncbi.nlm.nih.gov/) for the following alleles of the dwarfing genes: Rht-B1b, Rht-B1e, Rht-B1p and Rht-D1b. Also, the sequences of the wild-type alleles Rht-A1a, Rht-B1a and Rht-D1a were retrieved as controls. The Genbank accession numbers for used sequences are given in Supplementary material 1. Sequence alignment confirmed the presence of nonsense mutations in these allelic forms, which made it possible to start the development of CAPS and dCAPS markers (see Fig. 1).

A search was made for each nonsense mutation to identify restriction sites that would distinguish the target allele from all others, including wild-type ones. The BstV1I (GCAGC) restriction enzyme, unable to digest the mutant GTAGC site, was selected for Rht-B1b. Similarly, BstHHI (GCGC) became the restriction enzyme for the Rht-B1p detection (mutant site GCGT). On the contrary, the BstSFI restriction enzyme (CTRYAG) exclusively digested the mutant site (CTGTAG) contained in Rht-D1b. Thus, it was possible to develop such CAPS markers as CB1b/BstV1I, CB1p/BstHHI and CD1b/ BstSFI to identify the alleles Rht-B1b, Rht-B1p and Rht-D1b, respectively.

We failed to identify restriction sites at the location of the nonsense mutation in the Rht-B1e allele. Hence, the dCAPS marker dCB1e/HinfI was developed for it: the sequence of the reverse primer was modified so that the analyzed nucleotide, together with the 3′ end of the primer, formed a GATTC restriction site, providing an opportunity to distinguish this mutation from all other alleles by means of the HinfI restriction. The dCAPS marker dCB1b/Acc36I was additionally constructed to identify Rht-B1b (see Fig. 1).

When performing PCR under standard conditions, with the genomic DNA of bread wheat used as a template, we were unable to obtain specific fragments for the dCAPS markers and the CAPS marker CB1p/BstHHI (the data are not presented). We therefore applied the nested PCR method: the amplification products of the BF/VIR.B1R primers, flanking the region of localization of all analyzed point mutations in the Rht-B1 gene, were used as a template for the second round (see Fig. 1).

**Fig. 3.**
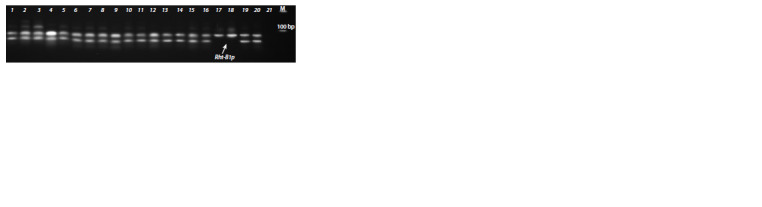
Identif ication of the Rht-B1p allele with the CAPS marker CB1p/BstHHI. The arrow points at genotypes with Rht-B1p, the PCR products of which were not restricted. Numbers designate accessions with different alleles of the Rht-B1 and Rht-D1 genes: 1, 2 – Hongdongmai (wild-type); 3, 4 – Krasota (Rht- B1b, Rht-D1a); 5, 6 – Knyazhna (Rht-B1b, Rht-D1a); 7, 8 – Grom (Rht-B1e, Rht-D1a); 9, 10 – Veda (Rht-B1e, Rht-D1a); 11, 12 – Triumph (Rht- B1i- 1, Rht-D1a); 13, 14 – Chinese Spring (wild-type); 15, 16 – Zheng 9023 (Rht-B1h, Rht-D1b); 17, 18 – Chris Mutant (Rht-B1p, Rht- D1a); 19, 20 – Atlas 66 (Rht-B1h, Rht-D1a); 21 – negative control (H2O). М – molecular marker 100 bp DNA Ladder (SibEnzyme).

The developed markers were tested on a set of bread wheat accessions with known alleles of the dwarfing genes, and all of them demonstrated high efficiency in differentiating the wild-type Rht-B1a, Rht-D1a and mutant versions Rht-B1b, Rht-B1e, Rht-B1p and Rht-D1b (see Fig. 2 and 3).

Concurrently, allele-specific primers retrieved from published sources were used to identify nonsense mutations in Rht-B1b, Rht-B1e, Rht-B1p and Rht-D1b compared to the wild type (Ellis et al., 2002; Pearce et al., 2011; Bazhenov et al., 2015). For this purpose, two pairs of primers were used for identification of each mutation: one of them detected the mutant version, while the other spotted the wild type and all other alleles. It was shown for Rht-B1b, Rht-B1e and Rht-D1b that the results of allele-specific PCR on the whole agreed with the data of CAPS and dCAPS analyses. However, identification of the wild-type Rht-B1a and Rht-D1a alleles with the primers BF/WR and DF2/WR2, respectively (Ellis et al., 2002), involved certain difficulties: poor reproducibility of results, and generation of weakly expressed fragments in forms with Rht-B1b and/or Rht-D1b (Supplementary material 2). In the case of Rht-B1p, allele-specific PCR under the conditions of this study turned out to be ineffective: after amplification with the Rht-B1p-F/R1 primers (Bazhenov et al., 2015), a specific product was generated both in the forms with mutant alleles and in those with the wild-type ones (see Supplementary material 2).

The study also employed five pairs of STS primers (Pearce et al., 2011; Li et al., 2013; Lou et al., 2016) as a tool for identifying mutations associated with the presence of a retrotransposon in the coding region (Rht-B1c) as well as with insertions in the promoter region (Rht-B1i-1) and the 5′ flanking region (Rht-B1h). The locations of these insertions are marked in the scheme of the Rht-B1 gene; it also shows primers for their detection (see Fig. 1, a).

**Fig. 4.**
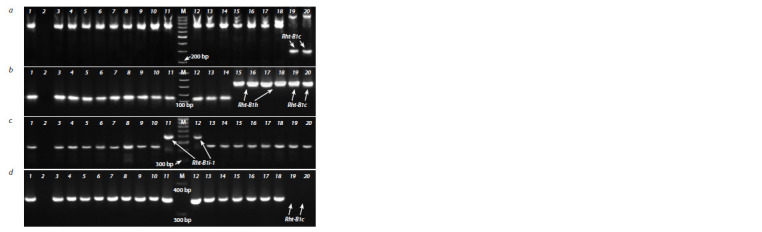
Identif ication of insertion-carrying alleles for the Rht-B1 gene using STS primers. a – PCR products of the Rht-B1c-F1/R1 primers specif ic to Rht-B1c; b – PCR products of the Rht-B1h-MF1/MR2 primers specif ic to Rht-B1h and Rht-B1c; c – PCR products of the B1i-MF2/MR2 primers specif ic to Rht-B1i-1; d – absence of PCR products of the BF/ VIR. B1R primers in genotypes with the Rht-B1c allele carrying a 2026 bp insertion. Numbers designate accessions with different alleles of the Rht-B1 and Rht-D1 genes: 1 – Krasota (Rht-B1b, Rht-D1a); 2 – negative control (H2O); 3, 4 – Knyazhna (Rht-B1b, Rht-D1a); 5, 6 – Grom (Rht-B1e, Rht-D1a); 7, 8 – Veda (Rht-B1e, Rht-D1a); 9, 10 – Chris Mutant (Rht-B1p, Rht-D1a); 11, 12 – Triumph (Rht-B1i-1, Rht-D1a); 13, 14 – Chinese Spring (wild-type); 15, 16 – Zheng 9023 (Rht-B1h, Rht-D1b); 17, 18 – Atlas 66 (Rht-B1h, Rht-D1a); 19, 20 – Tom Thumb (Rht-B1c, Rht-D1a). М – molecular marker 100 bp DNA Ladder (SibEnzyme).

Two pairs of primers were used for the Rht-B1i-1 allele (Lou et al., 2016): one of them (B1i-MF1/MR1) in the presence of an insertion produced a specific 330 bp fragment, while the other (B1i-MF2/MR2) amplified fragments of different sizes in genotypes with or without an insertion (see Tables 2 and 4, Fig. 4, c). Similarly, to detect the Rht-B1h allele, the Rht- B1h.F/R1 primers were used, resulting in a specific product of 247 bp, as well as the Rht-B1h.F/R2 primers, generating fragments of different sizes (see Tables 2 and 4; Fig. 4, b) (Li et al., 2013). Since Rht-B1h has a common insertion in the 5′ flanking region with Rht-B1c, the Rht-B1c-F1/R1 primer, specific for the retrotransposon sequence, was also used for their differentiation (see Tables 2 and 4, Fig. 4, a) (Pearce et al., 2011). Additional evidence of the presence of a retrotransposon may be found in the fact that no PCR products are generated in genotypes with this insertion during the first round of nested PCR with the BF/VIR.B1R primers: it can be explained by a big distance between the primers (see Fig. 4, d ).

**Table 4.**
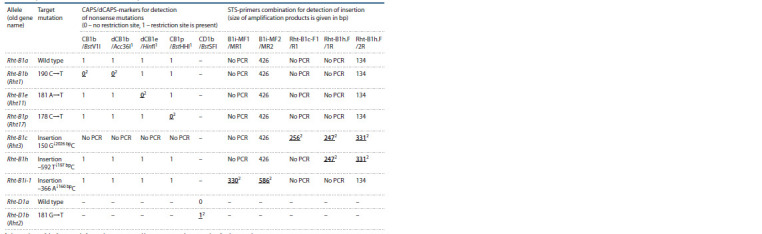
Marker prof iles for identifying alleles of the Rht-B1 and Rht-D1 dwarf ing genes using the system proposed in the present study 1 The products of the f irst round of nested PCR (50 times dilution) were used as a template for these markers. 2 Boldfaced and underlined are the amplif ication products, the presence/absence of which makes it possible to pinpoint the target alleles of the Rht-B1 and Rht-D1 genes.

Our testing of STS markers showed a complete concordance between the presence of their diagnostic fragments and the composition of alleles present in the studied genotypes (see Fig. 4). The accessions Atlas 66 and Zheng 9023 containing Rht-B1h yielded amplification products pointing to the presence of an insertion in the 5′ flanking region. In the accession Triumph carrying the Rht-B1i-1 allele, which increases plant height, an insertion in the promoter region was detected using molecular markers, and in Tom Thumb (Rht-B1c), a retrotransposon in the coding sequence and an insertion in the 5′ flanking region were found. It should be mentioned that when a retrotransposon was identified using the Rht-B1c- F1/ R1 primers, in addition to the formation of a fragment of the expected size, the emergence of nonspecific products of a larger size was observed in Tom Thumb (Rht-B1c) and in all other genotypes (see Fig. 4, a).

Assessing the system of the proposed molecular markers in its entirety, it should be kept in mind that it can be used to generate a marker profile for each of the studied alleles of the Rht-B1 and Rht-D1 genes, i. e., to get an unambiguous answer whether one of the abovementioned alleles of the Rht-B1 and Rht-D1 dwarfing genes is present in one or another genotype. Marker profiles for the alleles are presented in Table 4.

## Conclusion

As a result of this study, a system of molecular markers was proposed for the Rht-B1 and Rht-D1 dwarfing genes to identify the alleles most often used in bread wheat breeding. The system is based on the developed CAPS and dCAPS markers of nonsense mutations in these genes, which were previously detected by allele-specific PCR (Ellis et al., 2002; Pearce et al., 2011; Bazhenov et al., 2015, 2019). Five STS markers retrieved from published sources were used to identify insertions (Pearce et al., 2011; Li et al., 2013; Lou et al., 2016).

The CAPS and dCAPS markers were tested during the genotyping of bread wheat accessions from the VIR collection, containing the mutant Rht-B1b, Rht-B1c, Rht-B1e, Rht-B1h, Rht-B1i-1, Rht-B1p and Rht-D1b alleles as well as those of the wild-type. The tests showed complete concordance of the obtained results with the expected ones. The presence of Rht- B1b, Rht-B1e, Rht-B1p and Rht-D1b was also confirmed by allele-specific PCR with the primers widely used in research and breeding programs (Kurkiev et al., 2008; Pestsova et al., 2008; Divashuk et al., 2013; Li et al., 2013; Lou et al., 2016).

The main advantage of our molecular marker system lies in good reproducibility of results and their unambiguous interpretation. The CASP/dCAPS analysis faces no problems with controlling the PCR reaction success, because amplification products are formed in all genotypes, and differences between alleles are pinpointed after treatment with restriction enzymes. Besides, notwithstanding the high cost of restriction enzymes, CASP/dCAPS analysis is less expensive, since there is no need to perform two independent PCRs in several replications to detect each allele. The procedure is conducted employing standard PCR equipment and using agarose gel electrophoresis, so it can be carried out by small practiceoriented laboratories. When any new point mutations in the Rht-B1 and Rht-D1 dwarfing genes become known, a similar approach to the development of CAPS/dCAPS markers can be applied to identify them.

## Conflict of interest

The authors declare no conflict of interest.
